# The Relationship between High-reliability practice and Hospital-acquired conditions among the Solutions for Patient Safety Collaborative

**DOI:** 10.1097/pq9.0000000000000470

**Published:** 2021-09-24

**Authors:** Kelly H. Randall, Donna Slovensky, Robert Weech-Maldonado, Paul Sharek

**Affiliations:** From the * Ascension, Mo.; † University of Alabama at Birmingham, Ala.; ‡ Seattle Children’s Hospital, Division of General Pediatrics and Hospital Medicine, Department of Pediatrics, University of Washington, Seattle, Wash.

## Abstract

Theoretically, the application of reliability principles in healthcare can improve patient safety outcomes by informing process design. As preventable harm continues to be a widespread concern in healthcare, evaluating the association between integrating high-reliability practices and patient harms will inform a patient safety strategy across the healthcare landscape. This study evaluated the association between high-reliability practices and hospital-acquired conditions.

**Methods:**

Twenty-five pediatric organizations participating in the Children’s Hospitals Solutions for patient safety collaborative participated in this nonexperimental design study. A survey utilizing the high-reliability healthcare maturity model assessed the extent of implementing high-reliability practices at each participating site. We analyzed responses for each component and a composite score of high reliability against an aggregate measure of hospital-acquired conditions.

**Results:**

Of the 95 invited sites, 49 responded and 25 were included in the final results. There was a significant inverse relationship between the culture of safety component score and the Serious Harm Index (odds ratio [OR] = 0.63, 95% confidence interval [CI] 0.42–0.95, *P* = 0.03). There was no association between the overall high-reliability score (OR = 0.91, 95% CI 0.78–1.05, *P* = 0.19), the Leadership component score (OR = 0.97, 95% CI 0.70–1.33, *P* = 0.84), or the robust process improvement (RPI) component score (OR = 0.75, 95% CI 0.41–1.28, *P* = 0.26) and the Serious Harm Index.

**Conclusion:**

The integration of high-reliability principles within healthcare may support improved patient safety in the hospital setting. Further research is needed to articulate the breadth and magnitude of the impact of integrating high-reliability principles into healthcare.

## INTRODUCTION

Patients continue to experience serious preventable harm within the hospital setting.^1–4^ Patient safety researchers hypothesize that applying high-reliability principles to healthcare can transform organizations into settings that provide a safer patient care environment.^5–7^

Theoretically, the application of reliability principles can improve targeted patient safety outcomes by informing deliberate process design. The Institute for Healthcare Improvement indicates that strategically designing for high reliability is a critical factor of success.^8^ Hospitals were able to significantly decrease poor outcomes in the care of heart failure patients, patients admitted with pneumonia, surgical site infections, and return to smoking after acute myocardial infarction through such an approach.^8^ Few other studies exist that evaluate the direct association of high-reliability organization (HRO) principles to healthcare outcomes.

Based on the high-reliability principles of sensitivity to operations and reluctance to simplify,^7^ the use of care bundles resulted in a decreased occurrence of specific types of preventable harms known as hospital-acquired conditions (HACs). A care bundle is defined as a collection of best practices that, when reliably followed, can prevent an adverse event from occurring.^9^ Critical thinking and teamwork are encouraged by using the care bundles because reliably adhering to the care bundle requires collaboration and coordination of the entire team.^9^

Care bundles are prescriptive yet allow adaptation to the patient’s clinical situation within defined parameters. Bundles of best practices to prevent various HACs have been developed over time. Several studies have demonstrated a reduction in the rates of harm, such as central line-associated bloodstream infections and ventilator-associated pneumonia, when organizations reliably comply with these bundles.^10–19^ These studies are early “proofs of concept” that applying certain HRO principles in healthcare can indeed result in decreases in errors and patient harm.

To measure the overall impact of these improvement efforts, some organizations have adopted a composite measure of HACs, referred to as the Serious Harm Event (SHE) Index or the Preventable Harm Index.–^6,20,21^ This composite measure aggregates data from multiple preventable patient harms, providing a more comprehensive measurement of patient safety.^6,11,20,22^

The purpose of this study was to determine the association between high-reliability practices and HACs. This study builds on a previous study, which quantitatively assessed and described the extent and variability of integration of high-reliability practices among a collaboration of children’s hospitals using the High-Reliability Health Care Maturity Model (HRHCM).^5,23^

## METHODS

The Children’s Hospitals’ Solutions for Patient Safety (SPS) Network, established in 2011, is a collaboration of children’s hospitals working together to eliminate patient harm. The collaborative established a goal to reduce a defined set of HACs by 40%, originally over 2 years. The SPS network requires participating organizations to work on tactical quality improvement work by establishing and reliably performing care bundles to prevent HACs (such as the Surgical Site Infection prevention bundle and the Central Line-associated Blood Stream Infection prevention bundle^19^). As of January 1, 2015, the SPS collaborative comprised 95 children’s hospitals within the United States.^23^

Of the 95 pediatric organizations in the SPS collaborative at the time of the study, 49 responded to the high-reliability survey.^23^ Sixteen sites were excluded due to missing more than 10% of HAC data (13), organizational identification (2), or survey responses (1).

Of the remaining 33 sites, 8 were excluded based on their reporting data as part of a larger hospital system, resulting in a final sample of 25 children’s hospitals. Organizational descriptive and clinical staffing variable descriptive statistics are summarized in Table 1.

**Table 1.** Organizational Descriptive and Clinical Staffing Descriptive Statistics

	N	Frequency (%)	Mean	SD	Range
Bed size	25		335.5	122.1	73–595
RN hours per patient day	25		15.1	4.3	6.34–23.8
LPN hours per patient day	25		0.5	0.4	0–1.23
Medical staff hours per patient day	25		1.6	2.6	0–9.4
Employed physicians	25				
No		15 (60%)			
Yes		10 (40%)			

## DATA SOURCES

Participating sites reported monthly HAC data via web-based forms to a secure SPS database. SPS then provided these data to the investigators. The 2012 American Hospital Association (AHA) Annual Survey^24^ provided data for organizational descriptive and staffing variables.

We collected data from each organization on high-reliability practice based on a survey tool derived from the HRHCM.^23^ On behalf of SPS leadership, 2 authors (K.R. and P.S.) distributed the survey tool, with specific instructions, was distributed via email to representatives from each organization. SPS received all responses directly. Staff within the SPS then linked the survey data with respective organizational outcomes and hospital-characteristic data from the 2012 AHA Annual Survey.^24^ SPS staff de-identified and delivered these data to the principal investigator for this analysis.

## MEASURES

Patient safety outcome data available at the time of the study consisted of 8 HACs, each with standardized clinical and operational definitions established by HAC-specific expert panels through the SPS (Table 2). These expert panels utilized current published definitions to establish the operational definitions used in this study.

**Table 2.** Operational Definitions of HACs

HAC	Defining Agency	Patient Population	How Measured
CLABSI	NHSN	Patients of inpatient or observation status with a central line	Absolute number of CLABSI
CAUTI	NHSN	Patients of inpatient or observation status with an indwelling urinary catheter, excluding patient in the neonatal intensive care unit	Absolute number of CAUTI
OB AE	SPS defined	Expectant mothers admitted for delivery of the infant	Absolute number of OB AE
Falls	NDNQI	All patients of inpatient or observation status	Absolute number of falls with moderate injury or above
VAP	NHSN	All patients of inpatient or observation status who experienced mechanical ventilation	Absolute number of VAP
HAC	Defining agency	Patient population	How measured
ADE	NCC-MERP	All patients of inpatient or observation status	Absolute number of ADE
SSI	NHSN	Patients who underwent a spinal fusion, ventricular shunt placement or revision, or cardiothoracic surgery	Absolute number of SSI in patients who underwent a spinal fusion, absolute number of SSI in Patients who underwent a ventricular shunt placement or revision, absolute number of SSI in patients who underwent a cardiothoracic surgery
PU	NDNQI	All patients of inpatient or observation status	Absolute number of serious PU (stage III, IV, unstageable, deep tissue injury)

ADE, adverse drug event; CAUTI, catheter-associated urinary tract infection; CLABSI, central line-associated bloodstream infection; NCC-MERP, National Coordinating Council for Medication Error Reporting and Prevention’s Index for Categorizing Medication Errors; NDNQI, national database of nursing quality indicators; NHSN, National Healthcare Safety Network; OB AE, obstetrical adverse event; PU, pressure ulcer; SSI, surgical site infection; VAP, ventilator-associated Pneumonia.

The SHE index, a composite measure of preventable harm, was tracked and reported routinely by SPS. For this study, we defined the SHE index as the sum of events occurring between September 2014 and August 2015 for each of the HACs as specified in the following calculation:

The SHE index (total harms per month across the 25 included sites) was initially measured as a continuous variable with a mean of 75.3 harms per month per site, with a SD of 42.8, a minimum of 9, and a maximum of 162. Given the nonnormal distribution of the SHE data, the variable was transformed into a categorical variable by dividing the outcome into quartiles. The SHE index 25th percentile was 48, the 50th percentile was 62.6, and the 75th percentile was 91.5.

The authors used the HRHCM survey tool to quantify how high-reliability attributes were implemented in each organization. The survey tool utilized a 4-point measurement scale assigning a numerical value to each stage of high reliability along the continuum: beginning (1 point), developing (2 points), advancing (3 points), and approaching (4 points).^5,23^ We assessed performance in the components of leadership, safety culture, and RPI.^5,23^ The sum of the component scores resulted in an overall high-reliability practice score.^23^ To address minimal missing survey and patient safety outcome data in the included sites, we used mean substitution.

We determined individual and collaborative-wide maturity of high-reliability practice for each component measured in the high-reliability survey as well as an overall high-reliability practice composite. Table 3 provides a summary of the high-reliability practice scores.

**Table 3.** Descriptive Statistics of High-reliability Practice Scores

	N	Average (SD)	Minimum	Maximum
Overall high reliability	25	43.2 (6.5)	28	53
Leadership	25	19 (2.5)	11	23
Safety culture	25	15.1 (2.6)	10	20
RPI	25	8.9 (1.8)	5	12

## STATISTICAL ANALYSIS

All variables were evaluated to identify potential outlier data or data that exceeded 2.5 SDs given the study’s small sample size.^25^ We identified 2 outliers in the variable medical staff hours per patient day. The outlier values remained in the analysis, as there was no way to determine if the data were truly not representative of the population.^25^

Ordinal logistic regression models tested the association between the SHE index and overall high-reliability practice without controlling various organizational descriptive and clinical staffing variables. Additional ordinal logistic regressions tested the association between each component of high reliability (leadership, safety culture, and RPI) and the SHE index.

## RESULTS

When controlling for organizational descriptive and clinical staffing variables, the high-reliability practice safety culture component demonstrated a significant inverse relationship to the organization SHE index (odds ratio = 0.63, *P* = 0.03; Table 4). Hence, organizations had 37% lower odds of being in a higher SHE index quartile for each point increase in the safety culture component. There was no association between overall high-reliability practice, leadership, or RPI and the SHE composite outcome.

**Table 4.** Ordinal Logistic Regression Results on the Relationship between High-reliability Practice (Composite and Components) and SHE Index (N = 25)

	Odds Ratio	Significance (*P*)	Lower Confidence	Upper Confidence
High-reliability composite	0.91	0.19	0.78	1.05
Medical staff hours per patient day	0.75	0.16	0.50	1.12
Employed physicians	0.14	0.08	0.16	1.26
LPN hours per patient day	5.33	0.20	0.41	69.95
RN hours per patient day	1.34	0.03	1.04	1.73
Total number of licensed beds	1.02	0.01	1.01	1.04
Leadership composite	0.97	0.84	0.70	1.33
Medical staff hours per patient day	0.76	0.17	0.51	1.13
Employed physicians	0.24	0.17	0.32	1.82
LPN hours per patient day	4.92	0.23	0.34	66.28
RN hours per patient day	1.30	0.03	1.02	1.66
Total number of licensed beds	1.02	0.00	1.01	1.03
Safety culture composite	0.63	0.03	0.42	0.95
Medical staff hours per patient day	0.71	0.13	0.45	1.11
Employed physicians	0.07	0.03	0.00	0.75
LPN hours per patient day	12.03	0.08	0.71	204.03
RN hours per patient day	1.43	0.01	1.08	1.91
Total number of licensed beds	1.03	0.00	1.01	1.04
RPI composite	0.73	0.26	0.41	1.28
Medical staff hours per patient day	0.73	0.13	0.48	1.10
Employed physicians	0.14	0.09	0.02	1.41
LPN hours per patient day	7.61	0.15	0.48	119.56
RN hours per patient day	1.37	0.03	1.04	1.81
Total number licenced beds	1.02	0.01	1.01	1.04

The organizational descriptive variables bed size and registered nursing per patient day were significantly and positively related to the SHE index in the overall, leadership, safety culture, and RPI component models. The employed physicians’ organizational clinical staffing variable was significantly and inversely related to the SHE index only in the safety culture component model (OR = 0.07, *P* = 0.03). We summarize these results in Table 4.

We tested nonresponse bias by comparing participating sites to nonparticipating SPS sites that reported pediatric-specific data to the AHA Annual Survey of Hospitals.^24^ The comparison of the included and excluded sites revealed participating sites had significantly less licensed practical nursing hours per patient day, a significantly higher percentage of nonprofit status, and significantly more beds than participating sites. There were no statistically significant differences among the 2 groups in nursing hours per patient day, medical staff hours per patient day, employed physicians, or membership in a healthcare system.

## DISCUSSION

This research study sought to assess the association of high-reliability practice and patient safety outcomes, specifically preventable harms defined as HACs in children’s hospitals participating in the SPS collaborative. To our knowledge, this is the first study that tests the association between high-reliability practice as operationalized by the HRHCM^5^ and patient safety outcomes.

In this study, we found that the safety culture component of the HRHCM model was significantly inversely related to the SHE index. The safety culture component assessed trust, accountability, identification of unsafe conditions, strengthening systems, and assessment of the culture of safety.^5^ Specifically, the higher the safety culture component score, the lower the quartile of the SHE index.

This finding is consistent with previous studies that correlate culture of safety scores with isolated clinical outcomes.^26–29^ Our data expand on these studies by revealing an association between the perceived culture of safety and a collection of multiple HACs. This study suggests an association between safety culture and patient harm. This finding is supportive of the application of high-reliability principles within healthcare, at least the specific HRO element of establishing a robust safety culture.

This report expands on previous work exploring the relationship between the structured implementation of care bundles and specific HACs.^11,15–17,19^ This study adopted a broader view by evaluating the impact of high reliability on an aggregate measure of HACs, the SHE index. Previous studies demonstrating a reduction in HACs focused mainly on single HACs within specific units (ie, intensive care units), within either single organizations or among organizations coordinated through a statewide initiative or quality improvement collaborative.^12–19^

Regulatory agencies require that organizations evaluate the safety culture regularly but allow organizations to choose the evaluation strategy and frequency. Many organizational leaders and patient safety professionals within the SPS report building the culture of safety measurement into the patient safety program and mechanisms to remove barriers that undermine a culture of safety. Organization leaders should consider these data to inform strategic plans for continuous improvement, including strategies to detect patient harm and implement high-reliability practices. These data also support ongoing evaluation by regulatory agencies of the effectiveness of organizational leadership to continuously improve the safety culture.

This study revealed a lack of association between the remaining components of the HRHCM model (total score, leadership, and RPI) and patient harm. Multiple factors could explain these results. First, the results could be related to the small sample size of organizations and insufficient statistical power to determine any association between high-reliability practice and the SHE index. Second, there was limited variability within the high-reliability practice scores. Specifically, the range of high-reliability practice scores was small, potentially minimizing the model’s ability to detect any association between high-reliability practice and the SHE index. Third, the hypothesis may not have been supported due to the disruption reported within participating organizations. Only 5 of 33, or 15.2% of responding organizations, reported experiencing no significant organizational changes within the 2 years before the study. Disruption was reported in the form of significant leadership transitions, new or expansion of facilities, significant electronic medical record changes, additions of service lines, and mergers and acquisitions.^23^ These types of disruptions are associated with an increase in preventable harms, including HACs, and might have counterbalanced any positive impact of HRO practices.

There were several limitations of this study. First, the high-reliability framework we used has not been validated as a tool to assess high reliability. Despite this, it remains the most widely known HRO evaluation tool and carries with it face validity.^23^ Second, there could have been inaccuracies within the organizational survey response, most likely with a bias toward overestimating the site’s level of HRO. Despite the instructions provided regarding how to craft the survey’s organizational response, we validated that all organizations completed the survey as instructed.^23^ Third, the study’s sample size was small, appeared to favor large, free-standing children’s hospitals limiting our ability to extrapolate these findings to sites with alternative characteristics such as smaller children’s hospitals within adult hospitals. Fourth, we did not assess the extent of human factors expertise or the approach to knowledge management at each participating site, each of which may influence our primary outcome of HACs. Finally, variability in how participating sites ascertained HAC occurrence was likely. SPS encourages organizations to utilize active surveillance to detect HACs and report events through incident reporting systems voluntarily; however, there is no mandate for specific detection methods. Active surveillance reveals more events than voluntary reporting through incident reporting systems; however, not all organizations have the resources available to conduct active surveillance. The utilization of different detection methods may have had a significant impact on the SHE index and thus the associations to high reliability that we were exploring.

## CONCLUSIONS

In this study, we find that a higher maturity within the culture of safety domain of the HRHCM is associated with a lower total of HACs for organizations within the SPS collaborative. We were unable to identify a relationship between other HRHCM components and patient harm. Further studies are needed to understand better the associations between high reliability and patient safety in healthcare organizations.

## DISCLOSURE

The authors have no financial interest to declare in relation to the content of this article.
